# Microsporidial stromal keratitis and endophthalmitis in an immunocompetent patient

**DOI:** 10.1186/s12348-016-0099-7

**Published:** 2016-09-01

**Authors:** Arjun B. Sood, Matthew R. Debiec, Steven Yeh, Hans E. Grossniklaus, J. Bradley Randleman

**Affiliations:** 1 Department of Ophthalmology, Emory University, Atlanta, GA USA; 2 Emory Eye Center, Atlanta, GA USA; 3 Madigan Army Medical Center, Tacoma, WA USA; 4 Department of Ophthalmology, Keck School of Medicine of USC, Los Angeles, CA USA; 5 USC Roski Eye Institute, Los Angeles, CA USA

## Abstract

**Purpose:**

The purpose of this study is to report a case of microsporidial endophthalmitis after penetrating keratoplasty in a healthy patient and discuss the management.

**Methods:**

This is a case report.

**Results:**

A 69-year-old healthy male underwent penetrating keratoplasty for corneal scar secondary to herpes stromal keratitis. He presented with features of acute graft rejection 3 years later. After failure of medical management, a repeat full thickness keratoplasty was performed. Pathologic examination of the corneal specimen showed microsporidia. The patient then developed a chronic endophthalmitis, and a vitreous tap and injection followed by pars plana vitrectomy were performed. Pathologic examination of tissue showed microsporidia.

**Conclusions:**

Microsporidia are being increasingly identified as the cause of stromal keratitis. This is the first report of microsporidial endophthalmitis in a patient without underlying systemic illness.

## Findings

Microsporidia are a group of obligate intracellular spore-forming parasites that behave as opportunistic pathogens and cause ocular disease [1, 2]. Ocular manifestations include keratoconjunctivitis, stromal keratitis, scleritis, and rarely endophthalmitis [3, 4]. Here, we present a unique case of microsporidial stromal keratitis confirmed histopathologically masquerading as graft rejection. The patient subsequently developed endophthalmitis, which has not previously been reported in an immunocompetent patient.

### Case report

A 69-year-old male was referred to Emory Eye Center in June 2010 for herpetic stromal keratitis in the left eye. His past ocular history was notable for central retinal vein occlusion (CRVO) in the left eye 3 years earlier. He underwent multiple intravitreal injections of bevacizumab and triamcinolone for the treatment of CRVO-associated macular edema. He subsequently developed a suspected herpetic keratouveitis resulting in a dense central stromal scar and visual acuity of count fingers despite aggressive topical and oral antiviral therapy.

The patient underwent penetrating keratoplasty (PK) with extra-capsular cataract extraction and intraocular lens placement in July 2010. Pathologic examination of the corneal transplant showed microsporidial organisms. Visual acuity improved to 20/80 with use of rigid gas permeable contact lens, and the graft remained clear until March of 2013 when the patient presented with signs and symptoms of early graft rejection. Topical corticosteroids were utilized with no improvement, so repeat PK was performed in October 2014. Pathologic examination of the repeat PK specimen again showed microsporidia despite the absence of intraocular inflammation, hypopyon, or any other signs of clinical infection.

**Fig. 1:**
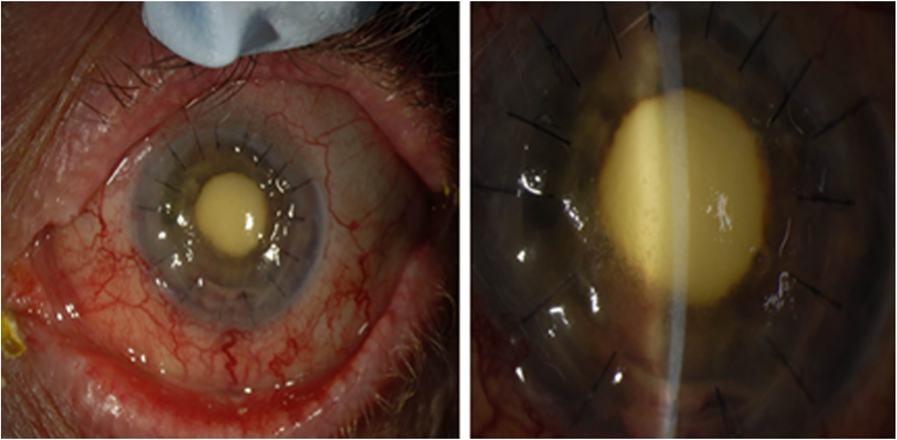
External photograph of left eye. The corneal sutures are intact. There is a *yellow* opacity overlying the pupil

Five weeks post-operatively, the patient presented with new onset floaters that had been present for 2 weeks. Visual acuity was light perception, and intraocular pressure was 17 mmHg. Anterior segment examination showed trace haze at the cornea graft-host interface, 1+ anterior chamber cells, a normal iris with a round and reactive pupil, and a well-positioned posterior chamber intraocular lens. There was no view of the posterior pole. B-scan ultrasound showed a presumed vitreous hemorrhage. The patient was lost to follow-up and presented again on post-operative week 9. Examination showed keratic precipitates, trace anterior chamber cell, and neovascularization of the iris. There was no view of the posterior segment due to a dense yellow opacity over the pupil (Fig. 1). B-scan ultrasound showed vitreous opacities, and the patient was diagnosed with presumed endophthalmitis. A vitreous tap and injection with vancomycin 1.0 mg/0.1 mL and ceftazidime 2.25 mg/0.1 mL were performed. A diagnostic and therapeutic pars plana vitrectomy was performed as the patient failed to improve. Examination of vitreous specimen and iris tissue biopsied at the time of vitrectomy showed microsporidia, consistent with a diagnosis of microsporidial endophthalmitis. Bacteria and fungi did not grow in culture. The patient was lost to follow-up and therefore did not undergo testing for the human immunodeficiency virus (HIV). To our knowledge, he was otherwise healthy and without underlying systemic disease.

### Pathologic findings

**Fig. 2:**
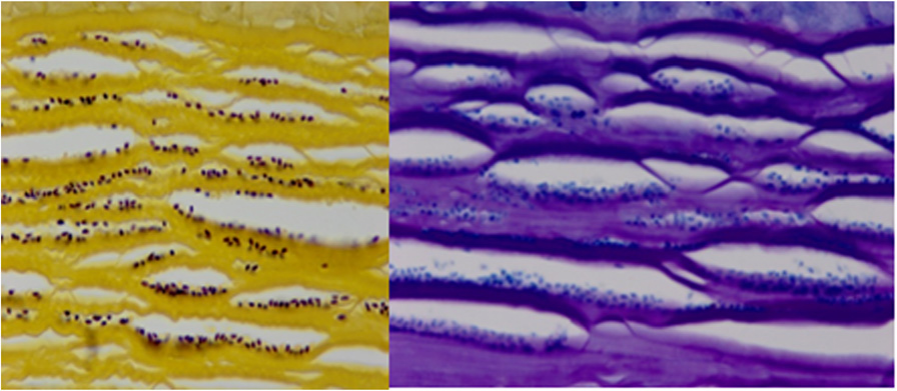
The superficial stroma showing organisms measuring 2 μm × 4 μm

**Fig. 3:**
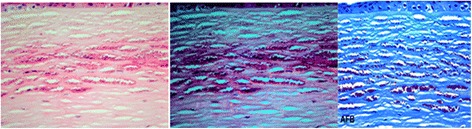
Intrastromal organisms without polarization (*left*) and with polarization (*middle*) and staining with AFB (*right*)

**Fig. 4:**
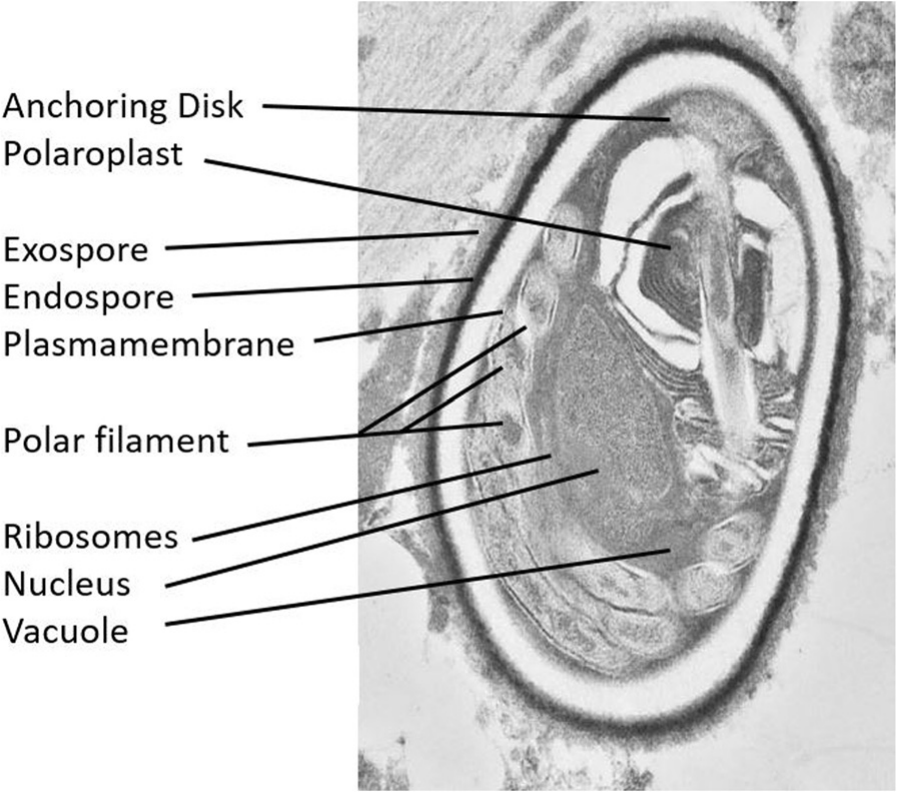
Transmission electron microscopy showing a 2 μm × 4 μm organism that contains an anchoring disk exospore, endospore, polaroplast, polar filament coils, nucleus, and ribosomes

Examination of the first penetrating keratoplasty specimen from 2010 showed a focally disrupted epithelium and Bowman’s layer. The superficial stroma contained a band of 2 μm × 4 μm organisms (Fig. 2). Examination of the second penetrating keratoplasty specimen showed intrastromal infiltrates or organisms as described for the first specimen. These organisms were polarized and stained with gram and AFB stains (Fig. 3). Examination by transmission electron microscopy showed 2 μm × 4 μm organisms that contained an exospore, endospore, plasma membrane, polaroplast, polar filaments, nucleus, and ribosomes (Fig. 4). Molecular PCR testing of the corneal sample was positive for Anncaliia algerae, part of the Nosema genera of microsporidia. Examination of the vitrectomy specimen with special stains demonstrates 2 μm × 4 μm AFB-positive organisms in the iris tissue. The final diagnosis for the corneal and vitrectomy specimen was microsporidiosis.

### Discussion

The case presented herein represents the first report of microsporidial endophthalmitis in a patient without underlying systemic illness at presentation. Microsporidia are opportunistic pathogens causing gastrointestinal, sinus, pulmonary, muscular, renal, and ocular diseases [1]. Classically, ocular manifestations include keratoconjunctivitis, corneal stromal keratitis, sclerouveitis, and rarely endophthalmitis [3, 4]. Keratoconjunctivitis, the most common primary ocular infection was described by Frieberg et al. in individuals with acquired immunodeficiency syndrome (AIDS) [5]. Clinical symptoms include sudden onset of unilateral pain, watering, and light sensitivity [6]. There may be an associated papillary or follicular conjunctivitis and the cornea will exhibit multiple, coarse, punctate epithelial lesions, which may evolve into a nummular keratitis. The disease resembles acute adenoviral keratoconjunctivitis or herpes simplex (HSV) keratoconjunctivitis [6]. Predisposing conditions include immunodeficiency, use of contact lenses, topical or systemic corticosteroids, trauma, and exposure to contaminated water. In India, most cases occur during the rainy season [7].

Microsporidia keratoconjunctivitis is generally a self-limited condition, especially in immune competent persons. A variety of drugs have been used as treatments, including fumagillin, propamidine, isethionate, and polyhexamethylene biguanide (PHMB), or with antifungal agents such as natamycin with or without systemic therapy with albendazole or itracanozole [8, 9].

Microsporidia stromal keratitis has rarely been reported [10]. The clinical diagnosis of stromal microsporidiosis is challenging and it may occur in immunocompetent contact lens wearers [11]. The initial clinical presentation mimics HSV stromal keratitis, and clinical signs vary depending on the stage of disease [6]. Predisposing factors include contaminated water, trauma, or contact with domesticated animals. The diagnosis is confirmed on corneal scrapings or tissue sections. The organisms measure approximately 2 μm × 4 μm, polarize, and stain with acid fast stains.

There is no definitive medical treatment for microsproidal stromal keratitis. Current literature suggests that the definitive treatment is excision of infected tissue and replacement with corneal tissue [6]. Recurrence after lamellar keratoplasty has previously been reported in two cases [6, 12]. However, recurrence after full thickness keratoplasty has not been reported previously. In our case, it is unclear as to whether there was a dormant infection that re-activated or a re-infection following the second corneal transplant.

There has been one previous case of microsporidial sclerouveitis diagnosed in a vitrectomy specimen [3]. There have been two previous cases of microsporidial endophthalmitis, both in immunocompromised individuals [4, 13]. In the first case, a 22-month-old boy with acute myelogenous leukemia developed superficial punctate keratopathy in both eyes, vitreous debris, and a dome-shaped retinal detachment. A vitrectomy demonstrated microsporidia by ultrastructural examination, and the infant was successfully treated with systemic albendazole; however, vision was limited to light perception with a persistent retinal detachment [4]. In the second case, a 15-year-old boy with idiopathic CD4+ T-lymphocytopenia developed epithelial keratitis, an anterior chamber reaction, and iris tumor in the left eye [13]. Biopsy of the mass showed *Encephalitozoon cuniculi* by transmission electron microscopy. The patient was treated with oral albendazole (400 mg bid) with improvement. Interestingly, that patient had CD4+ immunodeficiency, similar as to what occurs in AIDS.

Our patient’s social history was notable for residing on a rural farm. Upon further questioning during the course of initial graft failure, the patient endorsed using well water to bathe. Well water in rural areas is known to be contaminated with microsporidia. Therefore, we postulate that the mechanism of microsporidia graft infection in an otherwise healthy patient likely occurred by means of contaminated well water [7]. It is possible that the use of topical corticosteroids may have led to a dampening of the local immune response that would have otherwise eliminated microsporidia, but the precise reason for establishment of microsporidial infection is not clear.

### Conclusion

Microsporidia are being increasingly identified as the cause of corneal disease, and this case highlights the need to consider microsporidia in the differential diagnosis of stromal keratitis secondary to presumed herpetic eye disease. Microsporidia are fastidious organisms and are difficult to culture. Microscopic examination of tissue with appropriate staining can help make the diagnosis, and electron microscopy can identify the definitive genus. Medical management is not effective for most cases of microsporidial stromal keratitis, and surgical excision of tissue with transplant remains the gold-standard treatment.
